# Implications of Combined Exposure to Household Air Pollution and HIV on Neurocognition in Children

**DOI:** 10.3390/ijerph15010163

**Published:** 2018-01-20

**Authors:** Megan K. Suter, Catherine J. Karr, Grace C. John-Stewart, Laurén A. Gómez, Hellen Moraa, Duke Nyatika, Dalton Wamalwa, Michael Paulsen, Christopher D. Simpson, Niloufar Ghodsian, Michael J. Boivin, Paul Bangirana, Sarah Benki-Nugent

**Affiliations:** 1 Department of Epidemiology, University of Washington School of Public Health, Seattle, WA 98195, USA; msuter@uw.edu (M.K.S.); ckarr@uw.edu (C.J.K.); gjohn@u.washington.edu (G.C.J.-S.); 2 Department of Pediatrics, University of Washington School of Medicine, Seattle, WA 98105, USA; 3 Department of Environmental and Occupational Health Sciences, University of Washington School of Public Health, Seattle, WA 98195, USA; mpaulsen@uw.edu (M.P.); simpson1@u.washington.edu (C.D.S.); niloufar.ghodsian@gmail.com (N.G.); 4 Department of Global Health, University of Washington School of Public Health, Seattle, WA 98104, USA; gomezl4@uw.edu; 5 Department of Paediatrics and Child Health, University of Nairobi, Nairobi 30197, Kenya; moraahelen38@gmail.com (H.M.); dukenyatika@gmail.com (D.N.); dalton@africaonline.co.ke (D.W.); 6 Department of Psychiatry, Michigan State University, East Lansing, MI 48824, USA; Michael.Boivin@hc.msu.edu; 7 Department of Psychiatry, Makerere University College of Health Sciences, Kampala 7062, Uganda; pbangirana@yahoo.com

**Keywords:** 1-hydroxypyrene, carbon monoxide, neurocognition, polycyclic aromatic hydrocarbon, HIV, Sub-Saharan Africa, household air pollution, pediatric

## Abstract

Air pollution exposure and HIV infection can each cause neurocognitive insult in children. The purpose of this study was to test whether children with combined high air pollution exposure and perinatal HIV infection have even greater risk of neurocognitive impairment. This was a cross-sectional study of HIV-uninfected unexposed (HUU) and HIV-infected children and their caregivers in Nairobi, Kenya. We used a detailed neuropsychological battery to evaluate neurocognitive functioning in several domains. We measured caregiver 24-h personal CO exposure as a proxy for child CO exposure and child urinary 1-hydroxypyrene (1-OHP), a biomarker for exposure to polycyclic aromatic hydrocarbons (PAHs). Median 24-h caregiver CO exposure was 6.1 and 3.7 ppm for 45 HIV-infected (mean age 6.6 years) and 49 HUU (mean age 6.7 years), respectively; 48.5% of HIV-infected and 38.6% of HUU had caregiver 24-h CO levels exceeding the WHO recommended level. Median 1-OHP exposure was 0.6 and 0.7 µmol/mol creatinine among HIV-infected and HUU children, respectively. HIV-infected children with high urinary 1-OHP (exceeding 0.68 µmol/mol creatinine) had significantly lower global cognition (*p =* 0.04), delayed memory (*p =* 0.01), and attention scores (*p =* 0.003). Among HUU children, urinary 1-OHP and caregiver 24-h caregiver CO were not significantly associated with neurocognitive function. Our findings suggest that combined chronic exposure to air pollutants and perinatal HIV infection may be associated with poorer neurocognitive outcomes. High prevalence of air pollution exposure highlights the need to reduce these exposures.

## 1. Introduction

Worldwide, there are 2.1 million HIV-infected children under 15 years of age, 70% of whom reside in Sub-Saharan Africa (SSA) [1]. In this region, air pollution is a major public health concern [2]. Air pollution is a mixture of natural and man-made substances, including particulate matter (PM), carbon monoxide (CO), polycyclic aromatic hydrocarbons (PAHs), and sulfur dioxide [3]. Exposure to air pollution is associated with multiple morbidities, including pneumonia and cardiovascular events [4,5]. In SSA where 77% of households use solid fuel for cooking or heating, household air pollution (HAP) poses a significant health risk, and is a major contributor to ambient air pollution [2,6,7]. In 2012, HAP was attributed to 581,300 deaths in SSA [8]. The known implications of solid fuel use on HAP has resulted in efforts to promote use of cleaner fuels, such as kerosene [9]. However, there are few data to describe HAP and its impact on health outcomes in highly populous peri-urban communities in low and middle-income countries where biomass is less commonly used [9,10].

A growing evidence base implicates chronic early life air pollution in neurocognitive insult [11,12,13,14]. Significant brain developmental processes continue from prenatal life into early childhood and adolescence, making this entire period a critical window for central nervous system (CNS) development [15,16]. Environmental toxins in air pollution may cross the blood brain barrier, where they drive activation of microglia and astrocytes and trigger release of neurotoxic molecules [13,17]. Cellular damage may result in white matter changes that further impair brain development and function [13,18]. Recent studies have linked prenatal exposure to particulate matter (PM_2.5_), CO, and nitrogen oxide (NO_2_) in air pollution with impaired global cognition [19], visual spatial reasoning, short and long term memory [20], and fine motor skills in early childhood [20]. Prenatal PAH exposure is associated with deficits in nonverbal reasoning ability [21], developmental delay [22], reduced IQ [23,24], and verbal IQ [24,25]. Likewise, chronic early childhood exposure to higher levels of black carbon, NO_2_, and PAH in air pollution are associated with deficits in attention [26], verbal IQ [25], and learning ability [27,28]. 

Perinatal HIV infection can also cause a broad spectrum of cognitive impairment and neurologic disease, including progressive HIV-encephalopathy (PHE), neurocognitive delay and impaired cognition [29,30,31,32]. While the advent of antiretroviral therapy (ART) has substantially reduced the incidence of PHE [30], HIV-infected children on ART often have lower neurocognitive functioning compared to their uninfected peers and population norms [29,33,34,35]. HIV-infected children may manifest deficits in numerous domains including processing speed, memory, visual–spatial skills, global cognition, executive function, and reasoning [29,31,32]. Similar to environmental neurotoxicants, HIV neuropathogenesis involves both microglia and astrocytes and a neuroinflammatory molecular cascade that damages neurons [36]. White matter microstructural damage is common in HIV-infected children [37,38].

We hypothesize that HIV and chronic exposure to air pollution may impact neurocognition either through shared pathways, or through additive insult on existing damage. To date, no studies have investigated the impacts of air pollution on neurocognition in HIV-infected children. In this study, we tested whether HIV modifies the relationship between air pollution and cognition. We measured the magnitude of CO and PAH exposure among HIV-infected and uninfected children, and examined the relationship between these exposures and neurocognition in these two groups.

## 2. Materials and Methods

### 2.1. Participants and Recruitment

This study includes early-treated HIV-infected children and HIV unexposed uninfected (HUU) children and their caregivers. Participants in ongoing studies involving annual comprehensive cognitive and motor assessments were recruited for the Nairobi, Kenya-based Health Impacts of Household Air Pollution on Women’s Health and Child Survival (HAPK) Study. HIV-infected children had previously participated in the Optimizing HIV-1 Therapy Study (OPH03; NCT00428116), a trial designed to measure growth and development in infants randomized to continued or interrupted ART [39]. All enrolled children initiated ART during infancy (at <12 months of age) and had monthly study visits for up to 42 months. At the end of follow-up, children and their caregivers were invited to participate in a cohort study with extended follow-up and annual cognitive and motor assessments. From 2011–2013, HUU children were recruited from the Mathare North Maternal Child Health Clinic in Nairobi. Key eligibility criteria included: Age 5–12 years, and both biological mother and child confirmed HIV negative. Ethical approval for this study was obtained from the University of Washington (UW) Institutional Review Board (45269, 27 August 2013) and the University of Nairobi/Kenyatta National Hospital (KNH) Ethics and Research Committee (P23/6/2013, 27 November 2013).

### 2.2. Data Collection

At HAPK Study enrollment, study staff collected demographic information and information about typical cooking behaviors and fuel use using standardized questionnaires. To assess household air pollution (HAP), two home visits were conducted by study staff, 24-h apart, between December 2014 and December 2016. During the first study visit, staff conducted household surveys of cook-stove location and provided and installed air monitors. Twenty-four hours later, staff returned to collect air monitors, and administer questionnaires regarding caregiver adherence to wearing air monitors, and behavior related to air pollution exposure over the 24-h monitoring period. Additionally, study staff collected spot urine samples from caregivers and children for measurement of PAH metabolites. 

Caregiver personal CO exposure was measured during the 24-h monitoring period using Lascar electronic continuous CO monitors (EL-USB-CO). Caregivers were instructed to wear the monitors during waking hours and were asked to perform typical daily household activities. During the same 24-h monitoring period, household-level CO exposure was measured using a Lascar electronic continuous CO monitor (EL-USB-CO) hung in home cooking areas. 

PAH exposure was estimated by determination of a key PAH metabolite, 1-hydroxypyrene (1-OHP), in child urine samples. While PAH exposure is a mixture of compounds, pyrene is typically found in the mixture. Thus, its metabolite, 1-OHP, is often used as a proxy of PAH exposure from multiple sources [40]. Urinary metabolites are considered a useful biomarker for airborne PAH exposure [41]. Spot urine samples were stored at −70 °C on the same day as sample collection and were shipped to the University of Washington for metabolite analysis by high performance liquid chromatography with fluorescence detection. The analytical method was based on that reported by Chetiyanukornkul et al. [42], with modifications. The HPLC system was an Agilent 1100 series and the column was an Agilent Poroshell 120 SB-C18 (100 × 2.1 mm, 2.7 µm). Mobile phases were 10 mM sodium acetate (pH 5) and methanol. The lower limit of quantification was set at the concentration of the lowest calibration standard and all urine samples had 1-OHP greater than this. One-hydroxypyrene was measured in ng/mL, and values were creatinine-adjusted for dilution and expressed in µmol/mol of creatinine using the following formula:

(1) $$\frac{1-\mathrm{OHP}\;\mathrm{in}\;\mathrm{ng}/\mathrm{mL}}{\mathrm{creatinine}\;\mathrm{clearance}\;\mathrm{measured}\;\mathrm{in}\;\mu \mathrm{mol}/L}\times \frac{10^{6}}{218.3\;g/\mathrm{mol}}$$

A battery of neurocognitive assessments was performed by trained study staff, all with undergraduate degrees in psychology or graduate coursework in clinical psychology. Scripts for each assessment were translated from English to Kiswahili and back-translated to ensure accuracy. Tests were administered in the preferred language of the child, either Kiswahili or English. The Kaufman Assessment Battery for Children, Second Edition (KABC-II) was used assess the global cognition, short term and delayed memory, visual–spatial skills, learning, and non-verbal test performance [37]. The KABC has been used in Senegal [38] and Zaire [39], and in HIV-infected Ugandan children [40], and had good construct validity when administered to Ugandan children aged 7–16 years. The Test of Variables of Attention (TOVA) is a computer-based test that measures sustained attention based on visual stimuli. The TOVA has been used to characterize attention deficits in HIV-infected children [41] and children with a history of cerebral malaria [42] in Uganda [40]. The Behavior Rating Inventory of Executive Function (BRIEF) was used to measure executive function and consists of a caregiver-administered questionnaire. Previously, the BRIEF has been used in Ugandan children with HIV [43]. The Bruininks–Oseretsky Test of Motor Proficiency Brief Form (BOTMP-Brief Form), was used to assess overall motor proficiency. It has been previously used in HIV-infected populations ([34,38], pp. 309–332). Raw scores for each domain or scale were scaled and standardized using US norms. For ease of interpretation, scores are presented as z-scores.

### 2.3. Statistical Analysis

Analyses were stratified by child HIV-infected status because of our a priori hypothesis that the impacts of HAP may differ by infection status. Descriptive statistics for study population characteristics and neurocognitive outcomes (z-score) were calculated. Carbon monoxide (ppm) and 1-OHP (µmol/mol creatinine) variables were examined as both continuous and dichotomous variables. We dichotomized CO exposure (high/low) based on the 24-h WHO recommended limit of 6.11 ppm and PAH exposure (high/low) based on the cohort median for 1-OHP (0.68 µmol/mol creatinine). We calculated the arithmetic mean, standard deviation, median, interquartile range, minimum, and maximum 1-OHP and CO values. We compared 24-h mean CO and 1-OHP values by child HIV-infection status using 2-sample *t*-tests and compared medians with a non-parametric equality of medians test. We used chi-square tests to test whether high/low CO and 1-OHP exposure differed by child HIV infection status.

Continuous 1-OHP and CO were log_10_ transformed because the distributions of both variables were skewed. We evaluated cofactors for continuous CO and 1-OHP concentrations using univariate regression models (continuous cofactors), two-sample *t*-tests (dichotomous cofactors), and one-way ANOVA (cofactors with 3+ levels). We estimated the association between CO or 1-OHP concentrations and neurocognitive function using multivariate linear regression models. Confounders were selected a priori and included child age at time of neurocognitive testing and household monthly rent. All adjusted models were run with an exposure*HIV-infection interaction term and a likelihood ratio test was used to assess the statistical significance of the interaction term. Pearson correlation coefficients were used to evaluate the correlation between caregiver CO, household CO, child urine 1-OHP, and caregiver urine 1-OHP. All analyses were performed using Stata 14.0 (StataCorp, College Station, TX, USA) [43]. 

## 3. Results

### 3.1. Population Characteristics

Our sample included 49 HUU children and 45 HIV-infected children who had available data for either caregiver CO or urinary 1-OHP concentration. Caregiver CO data were available for 33 and 38 HIV-infected children and 38 HUU children, respectively, and child urinary 1-OHP data were available for 32 HIV-infected children and 43 HUU children. Mean age at time of neurocognitive testing was 6.6 years for HIV-infected children and 6.7 for HUU (Table 1). In both groups, the majority of caregivers were the child’s biological mother. Compared to HUU children, caregivers of HIV-infected children were less likely to be employed, less likely to be married, and reported a higher household monthly rent (mean 4105 vs. 2247 Kenyan Shillings), but had similar levels of education (9.5 vs. 9.0 years). Paraffin (kerosene) was the most common primary type of cooking fuel for both HUU children (76.5%) and HIV-infected children (45.5%). A significant proportion of the households for the latter group used propane as a primary fuel (34.1%).

### 3.2. Magnitude of HAP 

Mean 24-h caregiver CO was higher in HIV-infected children than in HUU children (11.59 ppm vs. 5.16 ppm, *p =* 0.04). The proportion of children with a caregiver 24-h mean CO exceeding the WHO recommended 6.11 ppm threshold, household mean 24-h CO values, and the proportion with household 24-h CO levels exceeding the WHO threshold did not differ significantly by HIV status (Table 2). Child 1-OHP concentration was similar in HIV-infected (0.9 µmol/mol creatinine) vs. HUU children (0.7 µmol/mol creatinine) (*p =* 0.6). The proportion of children with urine 1-OHP values exceeding the median was similar by HIV status (*p =* 0.7). 

### 3.3. Cofactors for HAP

In HIV-infected children, having an unemployed caregiver was significantly associated with higher urinary PAH (*p =* 0.01) and using a non-electric lamp for lighting was significantly associated with higher caregiver CO (*p =* 0.01). (Table 3). No other cofactors were significantly associated with HAP exposure. 

### 3.4. HAP and Neurocognition

In HIV-infected children, after adjustment for child age and household monthly rent, having a urine 1-OHP value exceeding the median (high 1-OHP) was associated with a global cognitive ability score that was −0.5 z-scores lower compared to children with a urine 1-OHP value less than the median (low 1-OHP) (β = −0.5, *p =* 0.04). High 1-OHP values were also associated with lower scores in the delayed memory (β = −0.7, *p =* 0.01), and attention (β = −1.1, *p =* 0.03) domains (Table 4a). In addition, HIV-infected children had an inverse linear relationship between increasing log_10_ 1-OHP concentration and attention scores (β = −0.8, *p =* 0.03) (Table 4b). After adjustment for child age and household monthly rent, caregiver 24-h CO concentration was not significantly associated with score in any domain among HIV-infected children. In HUU children, after adjustment for child age and household monthly rent, neither caregiver 24-h CO nor child urine 1-OHP concentration were associated with neurocognitive scores.

We observed a statistically significant interaction between high child urine 1-OHP concentration and HIV-infection in the delayed memory (β = −0.80, *p* = 0.03) and attention (β = −1.1, *p* = 0.02) domains, and a significant interaction between high caregiver CO concentration and HIV-infection in the attention domain (β = 0.21, *p* = 0.02). 

### 3.5. Correlations between Measurements of HAP

Correlations between measurements of HAP ranged from weak to strongly correlated (Table 5). Correlation was highest between household and caregiver CO (r = 0.70, *p* < 0.0001) and lowest for caregiver CO and child 1-OHP (r = 0.13, *p* = 0.4). 

## 4. Discussion

We examined the potential adverse neurocognitive health consequences of chronic exposure to common air pollutants (CO, PAH) among HIV-infected and HUU children in peri-urban Kenya. We hypothesized that impacts would be greater among HIV-infected children. Consistent with our hypotheses, we observed that HIV-infected children with higher 1-OHP in urine, a proxy for PAH exposure, had lower scores for global cognition, delayed memory and attention. Furthermore, there was a statistically significant interaction between high 1-OHP concentration and HIV-infection in the delayed memory and attention domains. In contrast, HUU children did not have differences in neurocognitive scores in relation to either their PAH or CO exposures.

Our results are consistent with those of previous epidemiological studies. Edwards et al. [21] observed an association between prenatal PAH levels and non-verbal intelligence at school age and Jedrychowski et al. [25] observed an association with lower verbal IQ in the same cohort. Similarly, in an urban New York cohort, Perera et al., found that higher prenatal PAH exposure was associated with lower IQ at age 5 [23]. We did not find any association between CO exposure and neurocognition, unlike Dix-Cooper et al. [20], who found associations between prenatal CO exposure and lower function in the visual–spatial integration, motor, short term memory, and long term memory domains. Our study differed in that it examined chronic childhood exposures, rather than prenatal exposure. It is possible that the exposures we measured were similar to earlier prenatal exposures in the same household and that observed effects reflect prenatal exposure. However, it also is plausible that both prenatal and postnatal exposures are associated with neurocognitive outcomes, given ongoing neuroplasticity during childhood [15,16].

To our knowledge, these data are the first to assess associations between postnatal exposure to HAP and neurocognitive outcomes in HIV-infected children. Our findings of associations between 1-OHP and multiple neurocognitive outcomes in HIV-infected but not HUU children suggests that the combination of HIV and environmental pollutants may have a detrimental impact on child neurocognitive outcomes. We and others have shown lower neurodevelopmental and neurocognitive functioning between HIV-infected compared with HUU children, despite antiretroviral therapy (ART) [29,30,32,33,35]. Similar to environmental toxins, HIV enters the CNS and triggers an inflammatory process in which small molecules, cytokines and chemokines disrupt neuronal function and cause neuronal cell death [14,36]. HIV-infected children may have pre-existing neurocognitive compromise that is worsened by exposure to environmental pollutants. Alternatively, or in addition, perinatal exposure to environmental toxins may also increase risk in HIV-infected children. In HUU, exposure to elevated levels of environmental pollutants did not have discernable impact, perhaps due to smaller magnitude of effects in this group. Mechanisms by which PAHs adversely affect the developing brain are not fully understood, but may involve endocrine disruption, binding of PAHs to placental growth factors, and oxidative stress [44,45,46]. Our data suggest that it would be useful to define mechanisms for synergies between HIV and PAH neurotoxicity and to decrease PAH exposures in HIV-infected children. 

In this Nairobi cohort, kerosene and propane, rather than biomass, were the most commonly reported cooking fuels, consistent with demographic surveys [47]. Kerosene is typically perceived by users as a cleaner alternative to biomass fuels [9], and propane is considered a low polluting fuel. However, multiple studies have linked kerosene use with high levels of emissions such as PM_2.5_ [9,28] and the associated health impacts [9]. An alarming 39% of children in our sample had levels of CO higher than WHO recommended limits for indoor levels. The mean maternal 48-h CO exposure in a Guatemalan cohort known for substantial wood smoke exposure is 3.8 ppm, while the mean 24-h caregiver CO in Nairobi families was 8.2 ppm. Similarly, mean 1-OHP levels were also high in our cohort, exceeding levels observed in other studies of young children. Mean 1-OHP levels in Ukrainian pediatric cohorts were 0.69 µmol/mol creatinine, and 0.34 µmol/mol creatinine, with the former corresponding to a cohort of children living near a steel mill [48]. The mean level in our cohort was 0.81 µmol/mol creatinine. The high levels of air pollution observed in our study underscore the need to further understand the key contributors to air pollution exposure in peri-urban cohorts, and the health impacts of these exposures, and whether interventions to decrease exposure to combustion byproducts can provide benefit. 

Strengths of this study include use of detailed neurocognitive assessment data, measurement of personal and household air pollution exposure (CO), and measurement of biomarkers for PAH exposures (1-OHP in urine). The neurocognitive assessments used in our study have been used previously in African and HIV-infected cohorts ([49,50,51,52,53,54,55,56], pp. 309–332). 

Our study has several limitations. First, this analysis was limited by a small sample size. Due to this study’s exploratory nature, we did not adjust for multiple comparisons. We were unable to control for some potentially important confounders, including nutritional factors, maternal IQ, psychosocial stimulation during early childhood, and exposure to other environmental toxicants. Our analysis only measured exposure to CO and PAH and we were unable to account for ambient air pollution exposure or other components of air pollution such as non-PAH PM_2.5_ constituents, nitrogen dioxide, metals, and ozone which also impact neurocognition [13].

The timing of collection of air pollution exposure data, which was performed when children were school-aged, may not reflect critical windows of neurodevelopment in the perinatal period. However, there are ongoing neurodevelopmental processes that continue into school age, which may be influenced by concurrent childhood exposures [11,16]. Additionally, we relied on a proxy measurement of child CO exposure; there are likely differences in the child’s versus the caregiver’s inhalation exposures due to differing minute ventilation, and the fact that school-age children are mobile. We employed an exposure assessment approach based on practical and cultural acceptability considerations. We can assume children spend a large proportion of their time (including sleeping time) in and around the home environment compared with other environments. Furthermore, we found strong correlation between caregiver and household CO measurements, suggesting compliance with wearing the monitors, and supporting the idea that caregiver CO is a reasonable proxy for household CO exposure. We would expect non-differential misclassification of CO exposure, which would bias our estimates toward the null. Another limitation of our exposure measurement is that use of the urinary 1-OHP biomarker does not allow us to differentiate the sources of PAH exposure, as it reflects exposure not just to HAP, but also to tobacco smoke, ambient air pollution, and dietary sources. 

HIV-infected children in our study were originally recruited for an RCT, and the unknown consequences of the trial intervention may be confounding our results. However, we did not find any differences in neurocognitive scores by randomization arm. Nonetheless, the impacts of this intervention on neurocognition should be carefully evaluated, though it is beyond the scope of this analysis. Another limitation of our study is the differing sample sizes between analyses. Children were included in our sample if they had either available caregiver CO data or urinary 1-OHP data. Thus, even though there was substantial overlap, the models assessing each exposure included slightly different samples (20 HIV-infected children and 32 HUU children who had both CO and 1-OHP data). While we did not find any meaningful differences in neurocognitive test scores or demographic characteristics between those with data for both exposures and those with data for either exposure, this could, in part, explain the differences between the 1-OHP and CO results. 

## 5. Conclusions

Despite limitations in timing of exposure assessment, use of a caregiver proxy CO measurement, and a modest sample size, our results provide further support of evidence that early life exposure to air pollutants such as PAH may compromise healthy neurocognition. The susceptibility among HIV-infected children, but not HUU children, is a novel and important observation. Given the large global population of children co-exposed to higher levels of air pollution and HIV in SSA, continued emphasis on characterizing and reducing risk factors for poorer neurocognitive health in the HIV-infected population is merited. Last, taking a multi-faceted interventional approach—combining biomedical interventions like ART with interventions to improve indoor air pollution—may be necessary to optimize neurocognitive outcomes for children with HIV in regions with high air pollution exposures.

## Figures and Tables

**Table 1:** Summary of study population sociodemographic characteristics and neurocognitive outcomes (z-score).

	HIV-Infected *n* = 45	HIV Uninfected *n* = 49
	*n* (%) or Mean (SD)	*n* (%) or Mean (SD)
**Sociodemographic Characteristics**		
Male sex	29 (64.4)	22 (44.9)
Child age at neurocognitive assessment (years)	6.6 (0.8)	6.7 (1.4)
Caregiver is biological mother	42 (93.3)	48 (98.0)
Caregiver is married	26 (57.8)	33 (70.2)
Caregiver is employed	11 (24.4)	16 (34.0)
Smoker in household	7 (15.6)	6 (12.8)
Cooks in living area	28 (62.2)	45 (93.8)
Garbage is burned nearby	12 (26.7)	16 (33.3)
Primary type of cooking fuel		
Wood	2 (4.6)	0 (0.0)
Propane	15 (34.1)	8 (17.0)
Charcoal	7 (15.9)	3 (6.4)
Paraffin (Kerosene)	20 (45.5)	36 (76.6)
Caregiver age (years)	33.2 (6.1)	31.1 (5.7)
Caregiver education (years)	9.5 (2.7)	9.0 (2.7)
Household people/room	3.4 (2.1)	4.4 (1.6)
Household monthly rent (Kenyan Shillings)	4105 (4801)	2247 (1311)
Time between neurocognitive assessment and air monitoring (months)	2.4 (3.6)	6.4 (3.9)
**Neurocognitive Function (z-score)**		
Global cognition	−1.9 (0.6)	−1.7 (0.8)
Short-term memory	−1.5 (0.8)	−1.3 (0.8)
Visual–spatial skills	−2.0 (0.6)	−1.8 (1.0)
Learning	−0.8 (1.0)	−0.9 (0.9)
Nonverbal test performance	−2.0 (0.7)	−1.9 (0.9)
Delayed memory	−1.0 (0.9)	−0.9 (0.8)
Executive function	0.2 (0.9)	0.1 (0.9)
Attention	−1.4 (0.8)	−1.1 (1.0)
Motor	−1.9 (0.9)	−1.6 (1.0)

Percentages indicated in the table are of those who had non-missing values for that variable. Missingness for all sociodemographic characteristics was <10%.

**Table 2:** Caregiver 24-h CO levels (ppm), household 24-h CO levels (ppm), and child urine 1-OHP (µmol/mol creatinine).

	**Caregiver 24-h CO, ppm**				**Caregiver 24-h CO Mean >6.11 ppm**
	***n***	**Mean (SD)**	**Median (IQR)**	**Range**	***n* (%)**
HIV-infected	33	* 11.6 (18.0)	6.1 (0.8, 13.2)	0.03, 83.0	16 (49)
HUU	38	* 5.2 (6.5)	3.7 (0.4, 7.1)	0.00, 31.6	12 (32)
	**Household 24-h CO, ppm**				**Household 24-h CO Mean >6.11 ppm**
	***n***	**Mean (SD)**	**Median (IQR)**	**Range**	***n* (%)**
HIV-infected	35	13.9 (19.4)	4.3 (1.2,27.0)	0.00, 95.2	16 (46)
HUU	40	9.2 (13.3)	3.8 (1.0,10.2)	0.00, 54.1	15 (38)
	**Child Urine 1-OHP (µmol/mol Creatinine)**				**High Child Urine 1-OHP**
	***n***	**Mean (SD)**	**Median (IQR)**	**Range**	***n* (%)**
HIV-infected	32	0.9 (0.7)	0.6 (0.4, 1.3)	0.05, 2.7	14 (44)
HUU	43	0.7 (0.5)	0.7 (0.4, 1.0)	0.07, 2.4	23 (53)

* Indicates statistically significant difference by HIV-infection status (*p* < 0.05). Means were compared with a *t*-test, medians were compared with a non-parametric test of equal medians.

**Table 3:** Cofactors for HAP exposure.

Cofactor	HIV-Infected				HIV Uninfected			
	Caregiver CO (ppm)		Urinary 1-OHP (µmol/mol Creatinine)		Caregiver CO (ppm)		Urinary 1-OHP (µmol/mol Creatinine)	
Household Characteristics	Geometric Mean (SD) or β (95%CI)	*p*	Geometric Mean (SD) or β (95%CI)	*p*	Geometric Mean (SD) or β (95%CI)	*p*	Geometric Mean (SD) or β (95%CI)	*p*
Type of cooking fuel								
Wood	0.8 (--)	0.1	2.6 (--)	0.3	--	0.8	--	0.2
Propane	3.4 (12.8)		0.5 (3.1)		3.4 (5.4)		0.4 (2.7)	
Charcoal	3.7 (2.9)		0.6 (1.8)		2.0 (3.4)		0.7 (1.2)	
Paraffin	2.7 (7.5)		0.6 (2.8)		1.9 (6.6)		0.6 (2.2)	
Smoker in household								
Yes	3.1 (7.8)	0.9	0.6 (1.8)	0.9	7.0 (1.4)	0.1	1.0 (1.6)	0.08
No	3.8 (7.2)		0.6 (2.9)		1.5 (6.3)		0.5 (2.3)	
Cooks inside living area								
Yes	3.3 (5.9)	0.9	0.5 (2.4)	0.2	2.0 (5.9)	0.8	0.6 (2.2)	0.6
No	3.1 (9.8)		0.9 (3.4)		2.4 (18.4)		0.4 (2.7)	
Non-electric lamp for lighting								
Yes	* 8.3 (4.0)	0.01	0.4 (2.9)	0.08	1.3 (7.3)	0.3	0.62 (2.5)	0.6
No	* 1.5 (8.4)		0.8 (2.4)		2.8 (5.4)		0.53 (1.9)	
Garbage burned nearby with smoke entering kitchen								
Yes	1.9 (3.5)	0.4	0.7 (2.6)	0.4	1.9 (7.6)	0.8	0.6 (2.2)	0.8
No	3.7 (8.7)		0.5 (2.8)		2.1 (5.9)		0.6 (2.2)	
**Socioeconomic Indicators**								
Caregiver employment status								
Employed	2.5 (13.3)	0.7	* 0.2 (3.1)	0.01	2.6 (5.0)	0.4	0.5 (2.6)	0.6
Unemployed	3.5 (6.3)		* 0.8 (2.4)		1.6 (7.9)		0.6 (2.2)	
Caregiver education (years)	−0.3 (−1.5, 1.0)	0.7	−1.5 (−3.4, 0.4)	0.1	0.2 (−0.9, 1.2)	0.7	−0.6 (−3.0, 1.9)	0.6
Household people per room	0.6 (−0.6, 1.9)	0.3	0.6 (−1.9, 3.1)	0.6	0.3 (−0.6, 1.1)	0.5	0.7 (−1.0, 2.4)	0.4
Household monthly rent	−774 (−3062, 1514)	0.5	−1243 (−2982, 495)	0.2	−183 (−760, 394)	0.5	619 (−597, 1834)	0.3

For continuous cofactors, *p*-values were calculated by comparing means of the log_10_ transformed HAP values with a *t*-test or one-way ANOVA. For continuous cofactors, coefficients and *p*-values were calculated by regressing the log_10_ transformed HAP values on the cofactor. * Indicates statistically significant difference between HIV = infected and HUU groups at α = 0.05.

**Table:** (**a**)

	HIV Infected			HIV Uninfected		
	Difference in z-Score by High vs. Low Child 1-OHP			Difference in z-Score by High vs. Low Child 1-OHP		
Neurocognitive Test Scores	*n*	β	95%CI	*n*	β	95%CI
Global cognition	31	* −0.5	−0.9, −0.03	39	−0.05	−0.6, 0.5
Short-term memory	31	−0.4	−1.0, 0.2	39	0.2	−0.4, 0.8
Visual–spatial skills	31	−0.4	−1.0, 0.1	39	−0.3	−1.0, 0.3
Learning	31	−0.6	−1.3, 0.003	39	0.2	−0.4, 0.8
Nonverbal test performance	30	−0.3	−0.8, 0.2	39	−0.5	−1.1, 0.2
Delayed memory	27	* −0.7	−1.2, −0.2	33	0.2	−0.4, 0.7
Executive function	31	0.05	−0.7, 0.8	40	0.0008	−0.6, 0.6
Attention	27	* −1.1	−1.7, −0.4	35	0.2	−0.5, 0.9
Motor	30	−0.3	−1.0, 0.4	40	0.2	−0.3, 0.7

* Indicates statistical significance at α = 0.05. All models adjusted for child age at time of neurological testing and household monthly rent.

**Table:** (**b**)

	HIV Infected			HIV Uninfected		
	Child 1-OHP			Child 1-OHP		
Neurocognitive Test Scores	*n*	β	95%CI	*n*	β	95%CI
Global cognition	31	−0.3	−0.8, 0.3	39	0.2	−0.5, 1.0
Short-term memory	31	−0.04	−0.7, 0.6	39	0.4	−0.4, 1.2
Visual–spatial skills	31	−0.4	−1.1, 0.2	39	0.008	−0.9, 0.9
Learning	31	−0.2	−1.0, 0.6	39	0.3	−0.5, 1.2
Nonverbal test performance	30	−0.3	−0.9, 0.3	39	−0.3	−1.2, 0.6
Delayed memory	27	−0.3	−0.9, 0.4	33	0.1	−0.8, 1.0
Executive function	31	0.06	−0.8, 0.9	40	0.02	−0.8, 0.8
Attention	27	* −0.8	−1.6, −0.07	35	0.04	−0.9, 1.0
Motor	30	−0.3	−1.1, 0.5	40	0.3	−0.4, 1.0

* Indicates statistical significance at α = 0.05. 1-OHP was log_10_ transformed. All models adjusted for child age at time of neurological testing and household monthly rent.

**Table:** (**c**)

	HIV Infected			HIV Uninfected		
	Caregiver 24-h CO			Caregiver 24-h CO		
Neurocognitive Test Scores	*n*	β	95%CI	*n*	β	95%CI
Global cognition	30	0.08	−0.2, 0.3	35	0.05	−0.3, 0.4
Short-term memory	30	0.04	−0.3, 0.4	35	0.1	−0.2, 0.4
Visual–spatial skills	30	0.05	−0.2, 0.3	35	−0.04	−0.4, 0.3
Learning	30	0.05	−0.4, 0.5	35	0.3	−0.05, 0.6
Nonverbal test performance	29	−0.3	−0.6, 0.04	35	−0.1	−0.5, 0.3
Delayed memory	26	0.1	−0.2, 0.5	28	0.1	−0.2, 0.5
Executive function	30	−0.4	−0.8, 0.07	35	−0.08	−0.5, 0.3
Attention	26	−0.05	−0.4, 0.3	30	−0.2	−0.7, 0.2
Motor	30	0.09	−0.3, 0.5	35	0.2	−0.1, 0.5

All models adjusted for child age at time of neurological testing and household monthly rent. CO was log_10_ transformed.

**Table 5:** Spearman correlation coefficient between log_10_ transformed measurements of household air pollution.

HAP Measurement	Household CO	Child 1-OHP	Caregiver 1-OHP
**Caregiver CO**	0.70 (*n* = 66, *p*<0.0001)	0.13 (*n* = 52, *p* = 0.4)	0.26 (*n* = 14, *p* = 0.38)
**Household CO**		0.24 (*n* = 57, *p* = 0.07)	0.50 (*n* = 14, *p* = 0.07)
**Child 1-OHP**			0.54 (*n* = 18, *p* = 0.02)
